# Passive exposure and perceptions of smoke-free policies in hospital and university campuses among nursing students: A cross-sectional multicenter study

**DOI:** 10.18332/tid/167390

**Published:** 2023-07-17

**Authors:** Marcela Fu, Yolanda Castellano, Kenza Laroussy, Antoni Baena, Mercè Margalef, Ariadna Feliu, Jordi Galimany-Masclans, Montse Puig-Llobet, Carmen Moreno-Arroyo, Raül Sancho, Albert Bueno, Antonio López, Joseph Guydish, Esteve Fernández, Cristina Martínez

**Affiliations:** 1 Tobacco Control Unit, Cancer Control and Prevention Program, Institut Català d’Oncologia, l'Hospitalet de Llobregat, Barcelona, Spain; 2 Tobacco Control Research Group, Epidemiology, Public Health, Cancer Prevention and Palliative Care Program, Institut d’Investigació Biomèdica de Bellvitge, l'Hospitalet de Llobregat, Barcelona, Spain; 3 Department of Public Health, Mental Health, and Maternal and Child Health Nursing, School of Nursing, Universitat de Barcelona, Barcelona, Spain; 4 Center for Biomedical Research in Respiratory, Madrid, Spain; 5 Department of Odonto-Stomatology, School of Medicine and Health Sciences, Universitat de Barcelona, Barcelona, Spain; 6 eHealth Center, School of Health Sciences, Universitat Oberta de Catalunya, Barcelona, Spain; 7 Department of Fundamental and Medical-Surgical Nursing, School of Nursing, Universitat de Barcelona, Barcelona, Spain; 8 Nursing Research Group, Digestive System, Diagnostics, Pharmacogenetics, Care Support and Clinical Prevention Program, Institut d’Investigació Biomèdica de Bellvitge, l'Hospitalet de Llobregat, Barcelona, Spain; 9 Nursing Care Management, Equip d’Atenció Primària Roses, Institut Català de la Salut, Girona, Spain; 10 Nursing Care Management, Equip d’Atenció Primària Valls Urbà, Institut Català de la Salut, Tarragona, Spain; 11 Philip R. Lee Institute for Health Policy Studies, University of California San Francisco, San Francisco, United States; 12 Department of Clinical Sciences, School of Medicine and Health Sciences, Universitat de Barcelona, l'Hospitalet de Llobregat, Barcelona, Spain

**Keywords:** attitudes, nursing students, public health, secondhand smoke exposure, smoke-free policies

## Abstract

**INTRODUCTION:**

Outdoor smoke-free regulations reduce exposure to secondhand smoke (SHS) and help to denormalize tobacco use. As future key agents in health promotion, nursing students’ attitudes should agree with tobacco-control policies. The objectives of this study were: 1) assess nursing students’ exposure to SHS in nursing schools, 2) explore their perceptions of compliance with the existing smoke-free regulations in acute-care hospitals; and 3) describe their support for indoor and outdoor smoking bans on hospital and university campuses.

**METHODS:**

This was a cross-sectional multicenter study conducted in 2015–2016 in all 15 university nursing schools in Catalonia, Spain. A questionnaire gathered information on SHS exposure, awareness of the smoke-free regulation in acutecare hospitals, and support for smoke-free policies in indoor and outdoor areas of hospitals and university campuses. Participants were nursing students attending classes on the day of the survey. We performed descriptive analyses and calculated adjusted prevalence ratios (APR) and 95% confidence interval (CI).

**RESULTS:**

Of 4381 respondents, 99.1% had seen people smoking in outdoor areas of their university campus, and 75.2% had been exposed to SHS on the campus (6.0% indoors and 69.2% outdoors). Nearly 60% were aware of the smoking regulation in place in acute-care hospitals. There was widespread support for smoke-free indoor hospital regulation (98.7%), but less support (64.8%) for outdoor regulations. Approximately 33% supported the regulation to make outdoor healthcare campuses smoke-free, which was higher among third-year students compared to first-year students (APR=1.41; 95% CI: 1.24–1.62), among never smokers (41.4%; APR=2.84; 95% CI: 2.21–3.64) compared to smokers, and among those who were aware of the regulation (38.4%; 95% CI: 1.37–1.75).

**CONCLUSIONS:**

Exposure to SHS on university campuses is high. Nursing students express low support for strengthening outdoor smoking bans on hospital and university campuses. Interventions aiming to increase their support should be implemented.

## INTRODUCTION

Exposure to secondhand smoke (SHS) is a health risk that causes avoidable morbidity and mortality^1^. For this reason, the World Health Organization, through the MPOWER package, assists countries to implement effective tobacco control measures to tackle the tobacco epidemic^2^. Some strategies have been more widely implemented, such as reducing both tobacco demand and SHS exposure in indoor public places^3,4^. Such policies have been mainly implemented in settings with a public or social role, such as hospitals and universities^5^.

Increasing awareness of the health consequences of active and passive smoking among nursing students is critical because they will play a key role in implementing smoking cessation strategies in their future practice^6,7^. They receive their education on university campuses but complete their clinical rotations in healthcare facilities; therefore, they are influenced by these two environments in which they learn and socialize. Increasing awareness can minimize the consequences of SHS exposure and denormalize tobacco smoking among future generations of nurses^7^.

Over the past decade, outdoor smoke-free hospital campuses among college students have been successful in reducing smoking initiation^8,9^, and increasing both perceptions of peers’ tobacco use and smoking norms^8^. However, few studies have assessed nursing students’ perceptions of smoking in indoor and outdoor areas of acute-care hospitals and university campuses.

Tobacco control law in Spain bans smoking in indoor public places and some outdoor public places, including acute-care hospital grounds^10,^ but it does not include university campuses. Considering that 29.7% of nursing students in Catalonia smoke^11^, outdoor areas near university entrances may concentrate smokers^12^, posing a health risk to non-smokers and promoting smoking normalization among future nurses.

The aims of this study were: 1) to assess SHS exposure in nursing schools as perceived by students; 2) to explore their awareness of the national smoke-free regulation in acute-care hospitals; and 3) to describe their opinions on the ban in indoor and outdoor areas of hospitals and university campuses.

## METHODS

### Design and participants

We conducted a cross-sectional study that included all university nursing schools in Catalonia (n=15). We contacted the deans of each school to request permission to conduct the survey and all of them agreed to participate in the study. The target participants were all nursing students enrolled in the 2015–2016 academic year, from the first to the fourth year (7660 nursing students). A non-probabilistic sample was obtained. To be included, participants had to: 1) be aged ≥18 years; 2) attend a regular class in a core subject on the day of data collection; and 3) provide written informed consent to participate. Core subjects were to be compulsory, so all students had to take them. They were selected at the discretion of the deans based on the number of students enrolled in the selected class, to ensure the highest possible participation. Students were not notified of the survey in advance. Additional details of the fieldwork have been described elsewhere^11,13^. The fieldwork was conducted between October 2015 and June 2016. In the class, all students were verbally informed about the objectives of the study by one of the researchers. After giving consent, they completed a paper-and-pencil questionnaire in an average of about 15 minutes. The study was approved by the Research Ethics Committee of the Bellvitge University Hospital and was conducted in accordance with the World Medical Association’s Code of Ethics for experiments involving human subjects (Declaration of Helsinki).

### Instrument and variables

An anonymous self-administered questionnaire was developed *ad hoc* and piloted in one of the universities, confirming its comprehension and applicability^11^. For the current analyses, we used variables related to compliance with smoking regulations, exposure to SHS, and agreement with smoke-free policies in acute-care hospitals and university campuses.

*Having seen people smoking on the university campus in the past week*

This was assessed by the item: ‘Please indicate how often you have seen people smoking on this campus in the past seven days’; done separately for indoor and outdoor areas. Responses were on a 5-point Likert scale response codes ranging from ‘never’ to ‘many times’. We dichotomized this variable by categorizing ‘never’ and ‘seldom’ as ‘no’ and everything else as ‘yes’ for ease of interpretation and simplicity of results.

*Exposure to SHS on the university campus in the past week*

This was assessed with the question: ‘In the past seven days, have you ever been exposed to tobacco smoke on this campus?’. Response options were ‘not exposed’, ‘exposed only indoors’, ‘exposed only outdoors’, and ‘exposed both indoors and outdoors’. The second and fourth categories were collapsed for analysis.

*Awareness of smoke-free policies in acute-care hospitals in Spain*

This was assessed with a multiple-choice question: ‘To the best of your knowledge, what is the current smoking policy in place in acute-care hospitals?’. Response options ranged from ‘smoking is allowed everywhere’ (the least restrictive) to ‘smoking is prohibited in all indoor and outdoor areas of the hospital, including the garden and walking or transit areas, the parking lot, etc.’ (the most restrictive and correct response). We dichotomized this variable into ‘aware of the policy’ and ‘not aware of the policy’. ‘Don't know’ responses were categorized as ‘not aware of the regulation’.

*Agreement with the prohibition of smoking*

This was assessed individually for indoors and outdoors in hospitals and in outdoor areas of university health sciences campuses and university campuses of any faculty. Each of the four questions had a 5-point Likert scale response options ranging from ‘strongly agree’ to ‘strongly disagree’. We collapsed the categories into ‘agree’ (‘strongly agree’ or ‘agree’) and ‘disagree’ (‘neither agree nor disagree’, ‘disagree’, and ‘strongly disagree’) with each statement to gain interpretability and simplicity of the results and to be able to run logistic regression models to identify predictors of agreement with each statement.

The main independent variables were: sex; year of nursing school (first, second, third, and fourth); type of university (public, private); and smoking status. Smoking status was categorized as: 1) smoker (either daily or occasional), 2) former smoker (person who smoked but has been abstinent for 6 or more months), and 3) never smoker^14^. For some analyses, we considered exposure to SHS in the last seven days on campus (yes, no), and being aware of the smoke-free policy in acute-care hospitals (yes, no), as independent variables.

### Statistical analysis

We calculated the proportions (%) and their corresponding 95% confidence intervals (CI) of self-reported exposure to SHS and the rest of the dependent variables. We estimated the factors associated with the agreement with smoking regulations in different locations using Poisson regression models with robust variance adjusted for all independent variables (sex, year of nursing school, smoking status, exposure to SHS, and awareness of the smoking policy). The variables used to fit the models were selected based on the theoretical framework, previous results in the literature, and data availability. The models provide prevalence ratios (PR) and 95% CI, which are the natural measure of association in cross-sectional studies, and indicate how many times a group agrees more with regulating smoking compared with a reference category group. The reference group was selected *a priori* on the assumption that it had the lowest agreement, to facilitate the interpretation of the results. We also applied weights to all analyses generated according to participation rates in each university. All tests were two-tailed, and the statistical significance was set at p<0.05. Analyses were performed with SPSS© 21.0 and STATA© 13 for Windows©.

## RESULTS

We obtained valid information from 4381 participants, representing 57.2% (4381/7660) of all nursing students enrolled in the 2015–2016 academic year in Catalonia and 98.5% (4381/4447) of all students attending the targeted classes. Approximately 84% were women and 58.4% were in their first or second academic year. Overall, 29.7% were current smokers and 57.2% had never smoked.

### Exposure to secondhand smoke on the campus

Approximately 17% of respondents had seen someone smoking in indoor areas, with significant differences by sex (men: 25.0%; women: 15.3%; p<0.001) and type of university (public: 20.1%; private: 14.1%; p<0.001). In contrast, 99.1% had seen someone smoking in outdoor areas, with no differences according to the variables studied (Table 1).

**Table 1:** Prevalence of seeing people smoking and being exposed to SHS on the university campus in the past 7 days among nursing students, ECTEC Study, Catalonia, Spain, 2015–2016 (N=4381)

*Characteristics*	*Have seen people smoking*						*Exposed to SHS*						
	*Indoors*			*Outdoors*			*Not exposed*		*Only outdoors*		*Only indoors plus indoors and outdoors*		
	*n*	*%*	*p ^a^*	*n*	*%*	*p ^a^*	*n*	*%*	*n*	*%*	*n*	*%*	*p ^a^*
Total	690	16.8		4306	99.1		1081	24.8	3008	69.2	260	6.0	
**Sex**			<0.001			0.908							0.002
Men	163	25.0		692	99.1		147	21.0	496	70.7	58	8.3	
Women	527	15.3		3614	99.1		934	25.6	2512	68.9	202	5.5	
**Year**			0.926			0.985							0.222
First	205	16.2		1331	99.1		351	26.1	908	67.6	85	6.3	
Second	177	16.8		1091	99.0		266	24.2	762	69.3	71	6.5	
Third	145	16.2		932	99.1		244	26.0	647	68.9	48	5.1	
Fourth	130	17.2		800	99.1		178	22.0	588	72.5	45	5.5	
**Type of university**			<0.001			0.426							<0.001
Public	371	20.1		1934	99.0		725	37.0	1109	56.7	123	6.3	
Private	319	14.1		2372	99.2		356	14.9	1899	79.4	137	5.7	
**Smoking status**			0.095			0.663							<0.001
Current	180	14.9		1263	99.1		181	14.2	1023	80.4	69	5.4	
Former	87	16.7		556	98.8		163	28.8	370	65.5	32	5.7	
Never	416	17.8		2447	99.1		722	29.3	1591	64.4	156	6.3	

SHS: secondhand smoke.

a Chi-squared test.

Six percent of participants reported being exposed to SHS in indoor areas (only indoors or both indoors and outdoors), while 69.2% reported being exposed only outdoors. Thus, 75.2% of the students were exposed to SHS somewhere on campus. Only 24.8% of the participants reported that they were exposed neither indoors nor outdoors (Table 1). There were some significant differences in the reporting of SHS exposure among students according to sex, type of university, and smoking status.

### Awareness of the smoke-free regulation in acute-care hospitals in Spain

Of all the participants, 59.3% were aware of the smoking regulation in acute-care hospitals. This awareness was higher among those in their final year of study (between 60% and 65%), those from a public university (62.5%) and among current and former smokers (around 62%) (Table 2).

**Table 2:** Prevalence of awareness of the smoke-free policy in acute-care hospitals in Spain among nursing students, ECTEC Study, Catalonia, Spain, 2015–2016 (N=4381)

*Characteristics*	*Aware of the regulation*		
	*n*	*%*	*p ^a^*
Total	2570	59.3	
**Sex**			0.597
Men	420	60.2	
Women	2150	59.1	
**Year**			<0.001
First	727	54.3	
Second	647	59.4	
Third	610	64.9	
Fourth	504	62.1	
**Type of university**			<0.001
Public	1222	62.5	
Private	1348	56.6	
**Smoking status**			0.008
Current	791	62.2	
Former	345	61.7	
Never	1413	57.4	
**Exposed to SHS^b^**			0.646
Yes	1911	59.0	
No	641	59.8	

a Chi-squared test.

b Exposed to secondhand smoke on the campus in the past 7 days.

### Agreement with regulating smoking in different places

Table 3 shows the association between students’ opinions about banning smoking in specific locations by independent variables. Most students (98.7%) agreed with banning smoking in indoor areas of hospital campuses, with no differences by independent variables. In contrast, 64.8% agreed with the current regulation banning smoking in outdoor areas of hospital campuses, especially among third-year students compared to first-year students (69.7%; APR=1.09; 95% CI: 1.03–1.16), never smokers (73.4%; APR=1.59; 95% CI: 1.49–1.69) and former smokers (67.4%; APR=1.44; 95% CI: 1.33–1.57) compared to current smokers, those who had not been exposed on the campus during the last seven days (69.6%; APR=1.04; 95% CI: 1.00–1.09), and those who were correctly aware of the smoke-free regulation in place in acute-care hospitals (75.4%; APR=1.54; 95% CI: 1.46–1.62).

**Table 3:** Prevalence of and factors associated with agreement to regulate smoking in hospitals and university campuses among nursing students, ECTEC Study, Catalonia, Spain, 2015–2016 (N=4381)

*Variables*	*Indoor hospital campuses*				*Outdoor hospital campuses*				*Outdoor university health science campuses*				*Outdoor university campuses of any faculty*			
	*n*	*%*	*APR*	*95% CI*	*n*	*%*	*APR*	*95% CI*	*n*	*%*	*APR*	*95% CI*	*n*	*%*	*APR*	*95 % CI*
Total	4306	98.7			2830	64.8			1435	33.0			1242	28.5		
**Sex**																
Men	688	98.3	1.00	0.98–1.01	463	66.0	1.04	0.99–1.11	235	33.7	1.05	0.93–1.20	218	31.1	1.15	1.03–1.29
Women (Ref.)	3618	98.8	1		2367	64.6	1		1200	32.8	1		1024	28.0	1	
**Year**																
First (Ref.)	1324	98.4	1		827	61.4	1		371	27.6	1		287	21.4	1	
Second	1097	99.2	1.01	0.99–1.02	726	65.7	1.05	0.99–1.11	352	31.9	1.12	0.99–1.28	316	28.6	1.31	1.15–1.49
Third	929	98.4	1.00	0.99–1.01	657	69.7	1.09	1.03–1.16	382	40.6	1.41	1.24–1.62	345	36.6	1.65	1.46–1.87
Fourth	799	98.8	1.00	0.99–1.01	520	64.0	1.02	0.96–1.09	279	34.5	1.24	1.07–1.45	244	30.0	1.39	1.18–1.65
**Smoking status**																
Current (Ref.)	1255	98.1	1		599	46.8	1		190	14.9	1		161	12.6	1	
Former	558	98.8	1.00	0.99–1.02	381	67.4	1.44	1.33–1.57	202	35.9	2.44	1.89–3.15	180	31.9	2.54	1.96–3.29
Never	2451	99.0	1.01	0.99–1.02	1819	73.4	1.59	1.49–1.69	1023	41.4	2.84	2.21–3.64	882	35.6	2.90	2.26–3.72
**Exposed to SHS^a^**																
Yes (Ref.)	3211	98.6	1		2061	63.2	1		1019	31.3	1		878	26.9	1	
No	1069	99.0	1.00	0.99–1.01	751	69.6	1.04	1.00–1.09	405	37.6	1.03	0.96–1.11	354	32.8	1.06	0.99–1.13
**Aware of the regulation**																
Yes	2532	98.5	1.00	0.99–1.01	1937	75.4	1.54	1.46–1.62	983	38.4	1.55	1.37–1.75	853	33.3	1.55	1.37–1.76
No (Ref.)	1739	98.8	1		875	49.6	1		443	25.2	1		381	21.6	1	

APR: adjusted prevalence ratio derived from Poison regression models with robust variance; adjusted for all independent variables studied (all included in the table). The variables were selected for their theoretical and statistical contribution based on previous results from the literature and our previous analysis^11^.

a Exposed to secondhand smoke on the university campus in the past 7 days.

Thirty-three percent of the participants were in favor of regulating smoking in outdoor areas of health science campuses. This support was higher among students in their final year compared to those in their first year (40.6% of students in their third year; APR=1.41; 95% CI: 1.24–1.62), also among never smokers (41.4%; APR=2.84; 95% CI: 2.21–3.64) and former smokers (35.9%; APR=2.44; 95% CI: 1.89–3.15) compared to smokers, and among those who were well aware of the regulation compared to those who were not well aware of it (38.4%; APR=1.55; 95% CI: 1.37–1.75). Regarding their agreement with the regulation of smoking in outdoor areas of all university campuses (not limited to health sciences), 28.5% agreed with the regulation; this support was higher among men (31.1%; APR=1.15; 95% CI: 1.03–1.29), those in their third academic year (36.6%; APR=1.65; 95% CI: 1.46–1.87) compared to those in their first year, never smokers (35.6%; APR=2.90; 95% CI: 2.26–3.72) and former smokers (31.9%; APR=2.54; 95% CI: 1.96–3.29) compared to current smokers, and among those who were aware of the regulation (33.3%; APR=1.55; 95% CI: 1.37–1.76) (Table 3).

We also performed a multilevel analysis using the type of university variable as the second level to assess the effect of the university as a confounding factor, but this variable was neither significant nor did it improve the fit of the models (data not shown).

## DISCUSSION

This study provides insight into compliance with indoor and outdoor smoke-free policies in hospitals and support for smoking bans on hospital and university campuses from the perspective of nursing students, two important aspects to explore due to their future role as tobacco control agents^15^. Almost all nursing students (99%) had seen people smoking outdoors on the university campus, and a significant proportion had seen someone smoking indoors (almost 17%). In addition, three out of four students had been exposed to SHS indoors or outdoors. These data suggest that indoor smoking is still a problem in these settings, and outdoor smoking is very prevalent, exposing non-smokers to the harms of SHS and normalizing tobacco use among students.

Although smoking has been banned in hospital campuses in Spain since 2011, four in ten nursing students were unaware of this national legislation. While almost all students support the current regulation banning smoking indoors, only three in five support such a regulation banning smoking outdoors in hospital campuses. In terms of their support for banning smoking on university campuses, only one-third of nursing students supported the adoption of this regulation on both all types of campuses and on health sciences campuses.

Regarding students’ awareness of the smoke-free policy in force on hospital campuses, our results are comparable to a previous study of hospitalized patients in Catalonia. In that study, 40% of patients were aware of the regulation; however, only a few had received verbal or written information about the policy (4.8% and 6.1%, respectively)^11^. In the current study, 60% of the students were aware of the smoke-free policy in acute-care hospitals; more specifically, students in their last year of training were slightly more aware of the policy than those in their first year. Students in their final year may have spent more time in hospitals for their clinical training. Unfortunately, we did not ask whether students were informed of the smoke-free policy before their placements. Nevertheless, a significant proportion of nursing students, even those in their final years of training, were unaware of the smoking ban in a setting where they were either going or had completed their training and where they were likely to be working shortly. It should be noted that patients who are hospitalized in acute-care hospitals consider that health professionals should be role models in tobacco cessation (75.3%) and that they should provide smokers with support to quit smoking (83.0%)^16^. Therefore, nursing students should be informed about smoke-free policies and be trained in how to provide smoking cessation services to meet the expectations of their future patients.

Regarding smoking on university campuses and exposure to SHS, our multi-center study is consistent with previous studies conducted by our group^5,12^. In one of these studies, we observed that young adults were more exposed to SHS in outdoor areas of higher education institutions than in outdoor areas of bars and terraces in Spain^12^. This could be because tobacco consumption is high (24.6%) among people aged 15–24 years^17^, and nursing students are not an exception; in fact, our data show that 29.7% of participants smoked daily or occasionally at the time of the survey^11^, so they smoke in different areas of their schools. Moreover, they spend most of their time in these environments. This finding has implications for national authorities and higher education institutions, as they are responsible for protecting staff and students from the hazards of SHS, both indoors and outdoors^18^. In Europe, the adoption of smoke-free policies on university campuses is rare, while primary and secondary schools are extensively regulated, both indoors and outdoors^19^. In contrast, in the United States, several foundations and non-governmental organizations have suggested that university administrators and stakeholders promote smoke-free policies, including: developing written policies; communicating them to students, faculty, and staff through multiple channels; gauging the level of support for such policies; and working with student, faculty, and staff associations to gain their support^20,21^. In the United States, 27% of college students benefit from tobacco-free campus policies^20^.

We observed some discrepancy between the percentages of respondents who saw someone smoking indoors (17%) and those who reported being exposed indoors plus indoors and outdoors (6%). One possible explanation for the observed results is that students may not be aware of their exposure. In fact, some studies have found that self-reported exposure to SHS underestimates actual exposure as measured by biological markers^22^. Another possible explanation is that students may have seen someone smoking, but if they were far away from them, they may not have felt exposed to SHS.

In our study, nursing students expressed low level of support for the implementation of more restrictive smoking policies on university campuses, particularly in outdoor areas. Therefore, it is necessary to raise their awareness of the risks of exposure to SHS and to communicate the benefits of having smoke-free outdoor environments at both the individual and global levels^23^. For nursing students in particular, it is essential to engage them in tobacco control strategies due to their future role as healthcare providers^24,25^. In this regard, a case study in the Netherlands showed that a ban on smoking in outdoor areas of a university was associated with increased support among students after its implementation (from 64.3% to 82.1%)^26^. These findings should encourage both higher education institutions and governments to adopt comprehensive smoke-free policies on campuses, regardless of the initial level of students’ support. Additional strategies following the implementation of smoke-free laws can support compliance. These may include signage, communication campaigns, and smoking cessation promotion^21^.

Comprehensive smoke-free policies reduce tobacco use among students and the university community and reduce SHS exposure^9,27^. In one study, smoke-free college campuses had a reduction in the number of cigarette butts on their campuses compared to colleges without such policies^28^. Smoking outdoors, and particularly near main entrances, can increase exposure to SHS in adjacent indoor areas^12,29^. In Catalonia, 29.7% of nursing students are smokers, of which 38% are occasional smokers^11^, so smoking cessation support is needed to create a smoke-free culture on university campuses and promote a healthy environment. Previous smoking cessation interventions for health profession students have shown that multicomponent interventions are effective^30,31^. These programs should include evidence-based smoking cessation treatments to prevent withdrawal symptoms and strategies to manage cravings and stress^30^. Unfortunately, this type of initiative is still rare in Spanish universities.

### Limitations

The cross-sectional nature of this study does not allow for causal interpretation. In addition, we did not validate SHS exposure by using objective measures such as biomarkers, so SHS exposure may be underestimated. Furthermore, because we only sought to investigate whether nursing students were exposed to SHS in different locations, the precise levels of exposure were not measured. Our data did not include the entire population of nursing students in Catalonia, as not all the students were present in class at the time of the survey. However, we surveyed almost 60% of the student population, and 98.5% of those who were invited to participate agreed to take part in the study^11^. The voluntary nature of participation may have introduced some selection bias, as those who agreed to participate may have been those who were more interested in tobacco control. Nevertheless, 98.5% agreed to participate, and the information provided was not uniformly favorable to smoke-free policies.

## CONCLUSIONS

Almost all nursing students had seen people smoking outdoors on the university campus, and 17% of them had seen someone smoking indoors, even though it is prohibited by law. In addition, around 40% of nursing students were unaware that smoking is prohibited in outdoor areas of acute-care hospitals in Spain, where they are likely to train and work. One in three nursing students supported the adoption of a smokefree outdoor policy on campus, and those in their final year of training and non-smokers were more supportive of this policy. Promoting more restrictive smoke-free policies in the higher education sector in Spain is crucial, as exposure to SHS is extremely high. There is an urgent need to improve the current Spanish legislation by extending smoke-free areas to university campuses and, more generally, to all adult education institutions. Some measures that can increase the university community’s support for these policies are promoting smoking cessation programs, communicating the dangers of tobacco and exposure to SHS, involving students in the creation of smoke-free campuses, and working with university associations to raise awareness of this hazard.

## Data Availability

The data supporting this research are available from the authors on reasonable request.

## References

[1] Carreras G, Lugo A, Gallus S (2019). Burden of disease attributable to second-hand smoke exposure: A systematic review. Prev Med.

[2] World Health Organization (2008). WHO Report on the Global Tobacco Epidemic, 2008: the MPOWER package.

[3] Anderson CL, Mons U, Winkler V (2020). Global progress in tobacco control: the question of policy compliance. Glob Health Action.

[4] Husain MJ, Datta BK, Nargis N (2021). Revisiting the association between worldwide implementation of the MPOWER package and smoking prevalence, 2008-2017. Tob Control.

[5] Martínez C, Méndez C, Sánchez M, Martínez-Sánchez JM (2017). Attitudes of students of a health sciences university towards the extension of smoke-free policies at the university campuses of Barcelona (Spain). Gac Sanit.

[6] Sreeramareddy CT, Ramakrishnareddy N, Rahman M, Mir IA (2018). Prevalence of tobacco use and perceptions of student health professionals about cessation training: results from Global Health Professions Students Survey. BMJ Open.

[7] Tavolacci MP, Delay J, Grigioni S, Déchelotte P, Ladner J (2018). Changes and specificities in health behaviors among healthcare students over an 8-year period. PLoS One.

[8] Bennett BL, Deiner M, Pokhrel P (2017). College anti-smoking policies and student smoking behavior: a review of the literature. Tob Induc Dis.

[9] Fallin A, Roditis M, Glantz SA (2015). Association of campus tobacco policies with secondhand smoke exposure, intention to smoke on campus, and attitudes about outdoor smoking restrictions. Am J Public Health.

[19] (2010). Ley 42/2010, de 30 de diciembre, por la que se modifica la Ley 28/2005, de 26 de diciembre, de medidas sanitarias frente al tabaquismo y reguladora de la venta, el suministro, el consumo y la publicidad de los productos del tabaco. In Spanish. Boletín Oficial del Estado.

[11] Martínez C, Baena A, Castellano Y (2019). Prevalence and determinants of tobacco, e-cigarettes, and cannabis use among nursing students: A multicenter cross-sectional study. Nurse Educ Today.

[12] Sureda X, Fernández E, Martínez-Sánchez JM (2015). Secondhand smoke in outdoor settings: smokers’ consumption, non-smokers’ perceptions, and attitudes towards smoke-free legislation in Spain. BMJ Open.

[13] Martínez C, Castellano Y, Laroussy K (2023). Knowledge, attitudes, and training in tobacco dependence and cessation treatment among nursing students in Catalonia (ECTEC Study): cross-sectional study. Int J Ment Health Addict.

[14] Husten CG (2009). How should we define light or intermittent smoking? Does it matter?. Nicotine Tob Res.

[15] Stanhope M, Lancaster J (2012). Public Health Nursing: Population-Centered Health Care in the Community.

[16] Martínez C, Castellano Y, Fu M (2020). Patient perceptions of tobacco control after smoke-free hospital grounds legislation: multi-center cross-sectional study. Int J Nurs Stud.

[17] Generalitat de Catalunya (2016). Departament de Salut. Enquesta de salut de Catalunya: Comportaments relacionats amb la salut, l’estat de salut i l’ús de serveis sanitaris a Catalunya.

[18] World Health Organization (2007). Protection from exposure to second-hand tobacco smoke: Policy recommendations.

[19] Martínez C, Martínez-Sánchez JM, Robinson G, Bethke C, Fernández E (2014). Protection from secondhand smoke in countries belonging to the WHO European Region: an assessment of legislation. Tob Control.

[20] Blake KD, Klein AL, Walpert L (2020). Smoke-free and tobacco-free colleges and universities in the United States. Tob Control.

[21] American Nonsmokers’ Rights Foundation (2009). Steps for enacting a smokefree college campus policy.

[22] Jeong BY, Lim MK, Yun EH, Oh JK, Park EY, Lee DH (2014). Tolerance for and potential indicators of second-hand smoke exposure among nonsmokers: a comparison of self-reported and cotinine verified second-hand smoke exposure based on nationally representative data. Prev Med.

[23] Hammerich A, El-Awa F, Latif NA, El-Gohary S, Borrero MDL (2022). Tobacco is a threat to the environment and human health. East Mediterr Health J.

[24] Bialous SA, Sarna L (2016). ISNCC Tobacco Position Statement. Cancer Nurs.

[25] Sarna L, Bialous S (2005). Tobacco control in the 21st century: a critical issue for the nursing profession. Res Theory Nurs Pract.

[26] Bommelé J, Troelstra S, Walters BH, Willemsen M (2020). Does support for smoke-free outdoor spaces increase after implementation?: A case study of a Dutch research center’s smoke-free campus transition. Tob Prev Cessat.

[27] Lupton JR, Townsend JL (2015). A systematic review and meta-analysis of the acceptability and effectiveness of university smoke-free policies. J Am Coll Health.

[28] Lee JG, Ranney LM, Goldstein AO (2013). Cigarette butts near building entrances: what is the impact of smoke-free college campus policies?. Tob Control.

[29] Martínez C, Guydish J, Robinson G, Martínez-Sánchez JM, Fernández E (2014). Assessment of the smoke-free outdoor regulation in the WHO European Region. Prev Med.

[30] Pardavila-Belio MI, García-Vivar C, Pimenta AM, Canga-Armayor A, Pueyo-Garrigues S, Canga-Armayor N (2015). Intervention study for smoking cessation in Spanish college students: pragmatic randomized controlled trial. Addiction.

[31] Vitzthum K, Koch F, Groneberg DA (2013). Smoking behaviour and attitudes among German nursing students. Nurse Educ Pract.

